# Enhancing Sleep and Mental Health: Longitudinal, Observational, Real-World Study From a Digital Mental Health Platform

**DOI:** 10.2196/83492

**Published:** 2026-04-27

**Authors:** Kristen M Van Swearingen, Komal Kumar, Benyetta High, Sara M Levens, Neha Chaudhary, Jessica R Watrous, Sara J Sagui Henson

**Affiliations:** 1 Department of Psychological Science, Health Psychology Doctoral Program, University of North Carolina at Charlotte, 9201 University City Blvd Charlotte, Charlotte, NC, 28223, United States, 1 704-687-8622; 2 Modern Health, San Francisco, CA, United States

**Keywords:** sleep, mental health, burnout, longitudinal, observational, real world, digital health, platform, workplace, employees, depression, anxiety, exhaustion, cynicism, efficacy, prospective, therapy, coaching, psychoeducation, well-being

## Abstract

**Background:**

Poor sleep is closely linked to mental health challenges and workplace burnout. Mental health and workplace stressors can impair sleep, while good sleep quality supports cognitive and emotional resources to cope with daily challenges. Despite positive outcomes of maintaining good sleep, many people struggle to get enough restorative sleep at night. Given the bidirectional relationship between sleep and mental health, evidence-based digital mental health solutions may offer an accessible and scalable approach to improving sleep quality.

**Objective:**

This study examines whether engagement with an employer-sponsored, multimodal digital mental health platform is associated with improvements in sleep quality over time, and whether changes in sleep quality are associated with concurrent changes in mental health and burnout outcomes.

**Methods:**

This 12-month prospective, observational study followed working adults who were newly registered to an employer-sponsored digital mental health platform (Modern Health). The platform leveraged technology (mobile and web) to connect employees with comprehensive provider-led and self-guided care through therapy, coaching, on-demand digital resources, and group psychoeducational sessions. Participants [N=578; 61.1% (n=353) women; mean age 33.88, SD 8.73 years; 40.3% (n=233) people of color] completed measures of self-rated sleep quality, depression, anxiety, and burnout (exhaustion, cynicism, and professional efficacy) at baseline and after 3 and 12 months of accessing the platform. Upon registering for the platform, participants were given an initial care recommendation, but could flexibly engage in any combination of services. Participants in this study engaged with at least one care modality, including therapy, coaching, psychoeducation sessions, and self-guided mental health resources. We examined perceived sleep quality and associations with other study variables at baseline, changes in perceived sleep quality over time, and whether changes in sleep quality correlated with concurrent changes in mental health and burnout.

**Results:**

At baseline, 42% (243/578) reported poor sleep quality and were more likely to have higher levels of depression, anxiety, and burnout. A generalized linear mixed-effects model showed that each additional month of platform access was related to an increased odds of having good sleep quality by 3.7% (*P*=.02). Linear mixed-effects models found that higher sleep quality over time was associated with lower depression, anxiety, exhaustion, cynicism, and efficacy (all *P*<.001). Among participants reporting poor sleep quality at baseline, 44% (62/141) reported good sleep quality at 12 months. Within this subgroup, paired sample *t* tests showed significant reductions in depression (−48.3%) and anxiety (−38.3%), and increased cynicism, burnout, though cynicism levels remained below the cutoff for high burnout (23.9%; all *P* <.01).

**Conclusions:**

Use of an employer-sponsored digital mental health platform was associated with meaningful improvements in self-reported sleep quality over 12 months. These gains were associated with significant reductions in depression, anxiety, and burnout symptoms, highlighting broader well-being benefits of comprehensive mental health care.

## Introduction

Sleep is a fundamental process for regulating our overall health and well-being [1-3]. Consequences of poor sleep include daytime impairment [4], diminished ability to manage challenges effectively [5], and negative impacts on mental health [6]. As such, poor sleep is commonly linked to a range of mental health challenges [7,8] and is often noted as a prominent, distressing symptom of conditions like anxiety and depression [8,9].

Poor sleep quality and mental health concerns share a bidirectional relationship. For instance, getting poor sleep due to a variety of environmental, situational, social, and psychological factors [10-12] can negatively impact mood and increase worry the following day [13,14]. Additionally, it can impair attention, decision-making, and concentration, all of which are essential for navigating daily challenges [15]. Consequently, poor sleep quality can reduce one’s capacity to effectively cope with stressors [5,16] and regulate the negative emotions that may result from impaired coping [17,18]. At the same time, individuals with elevated mental health symptoms tend to experience racing thoughts that can disrupt their sleep [19-23]. This bidirectional cycle can contribute to the development or exacerbation of mental health challenges [24].

Poor sleep quality not only impacts mental health, but the impact also extends to well-being in the workplace. Sleep supports the restoration of cognitive and emotional resources that are essential for tackling workplace challenges [15,17,25]. Consequently, poor sleep quality is linked to greater workplace stress across a range of occupations [26]. When workplace stress is not adequately managed, it can intensify to burnout [25]. Burnout encompasses a range of symptoms including emotional exhaustion, detachment from one’s work, and a diminished sense of accomplishment [27]. Improving sleep quality can help individuals to better manage their workplace stress and protect against feelings of burnout [28].

As sleep is interconnected with mental health and burnout, addressing either part of this relationship may help to effectively break the harmful cycle. For example, when people feel more mentally well and engaged at work, their sleep quality may improve, and when they achieve better quality sleep, they may better regulate their mood and contribute to productive and meaningful work. Mental health interventions such as cognitive behavioral therapy [29] and mindfulness-based therapies [30] have shown promise in improving sleep quality, even when sleep was not the primary focus of treatment. However, only around a quarter of adults in the United States receive the necessary treatment for a mental health condition [31].

Digital mental health solutions offer a promising approach to increasing access to evidence-based interventions to improve sleep quality. Digital mental health is an umbrella of services, such as telehealth, digital therapeutics, online group therapy, chat-based care, and digital self-guided content, that are delivered through online platforms, mobile apps, or wearables [32-35]. There are currently several digital health solutions available specifically for sleep, such as digital cognitive behavioral therapy for those with clinical insomnia (d-CBTi) [36]. However, many individuals experience subclinical sleep issues, and even the mere perception that our sleep quality is poor can have a significant negative effect on emotional well-being and daily functioning [37-39]. Therefore, solutions beyond d-CBTi that effectively improve sleep may have broader public health implications.

Digital health platforms that offer comprehensive mental health services and are multimodal (eg, offer services across multiple care modalities) may improve sleep outcomes by addressing the underlying mental health factors that can affect sleep. For instance, one study found that adolescents receiving nonsleep-related digital mental health services experienced improvements in sleep quality as their mental health symptoms improved [40]. This evidence is promising, but more research is needed to evaluate the broader impact among the general population. Additionally, it is essential to examine digital mental health platforms that take a holistic care approach, expanding the range of services and care modalities to support users with varying health and well-being needs.

This study addresses a significant gap in the literature by examining the influence of engagement in multimodal employer-sponsored digital mental health services on sleep quality and associated mental health and burnout outcomes over time. The platform uses technology and a stratified care model that takes into account employees’ clinical needs and preferences and connects them with the right level of care. This care includes a global network of licensed clinicians and certified coaches delivering one-on-one telemental health care, support, and group psychoeducation sessions, and an extensive on-demand library of digital resources. All care modalities are evidence-based, culturally centered, and focus on five domains of health and well-being: emotional, physical, social, professional, and financial health. Evaluating the impact of a platform that addresses multiple dimensions of well-being through scalable, evidence-based multimodal care could provide insights for population-level sleep solutions.

In this study, we investigated how changes in sleep quality after engagement with a comprehensive digital mental health platform for 3- and 12-month periods were associated with changes in mental health concerns (depression and anxiety). Additionally, we investigated how changes in sleep quality across these timepoints influenced aspects of organizational burnout, including exhaustion, cynicism, and professional efficacy.

Our first aim was to analyze baseline associations between sleep quality, mental health, and workplace burnout among employees newly registered for the platform to understand the risk factors and correlates of poor sleep. Given the bidirectional relationships among these variables, we hypothesized that poor sleep quality would be associated with worse depression and anxiety symptoms, and higher levels of burnout. Our second aim was to examine longitudinal changes in sleep quality over time among employees engaging with the digital mental health platform across 3 and 12 months. We hypothesized that individuals would improve their sleep quality after engaging with the platform. Our third aim was to explore whether changes in sleep quality over time were associated with concurrent changes in mental health symptoms and workplace burnout. We hypothesized that as an individual’s sleep quality improved, they would also experience improvements in their depression and anxiety symptoms and reduce their feelings of burnout.

## Methods

### Study Design

This was a 12-month prospective, observational study of adults from multiple workplace organizations who had newly registered for an employer-sponsored digital mental health benefits platform (Modern Health). Participants were eligible if they (1) were 18 years or older; (2) had access to a smartphone, tablet, or computer; (3) were employed by companies partnered with the mental health platform; and (4) had indicated they were planning to start care (by either matching with a therapist or coach, or engaging with one piece of digital content). Data were collected at 3 timepoints: baseline (after registration) and at 3 and 12 months following registration. Data were collected between September 2021 and March 2023.

### Ethical Considerations

The study was conducted in accordance with the Declaration of Helsinki and approved by the Western Clinical Group Institutional Review Board (protocol code 1316167) on August 20, 2021. All participants provided informed consent to participate in the study. Participant privacy was protected by deidentifying all data collected. Data were securely stored on secure, encrypted servers via Box that were only accessible by the researchers. Participants were offered a US $25 digital gift card as compensation for completing each survey (up to US $75 total for completing surveys at all 3 timepoints).

### Procedure

**Figure 1.**
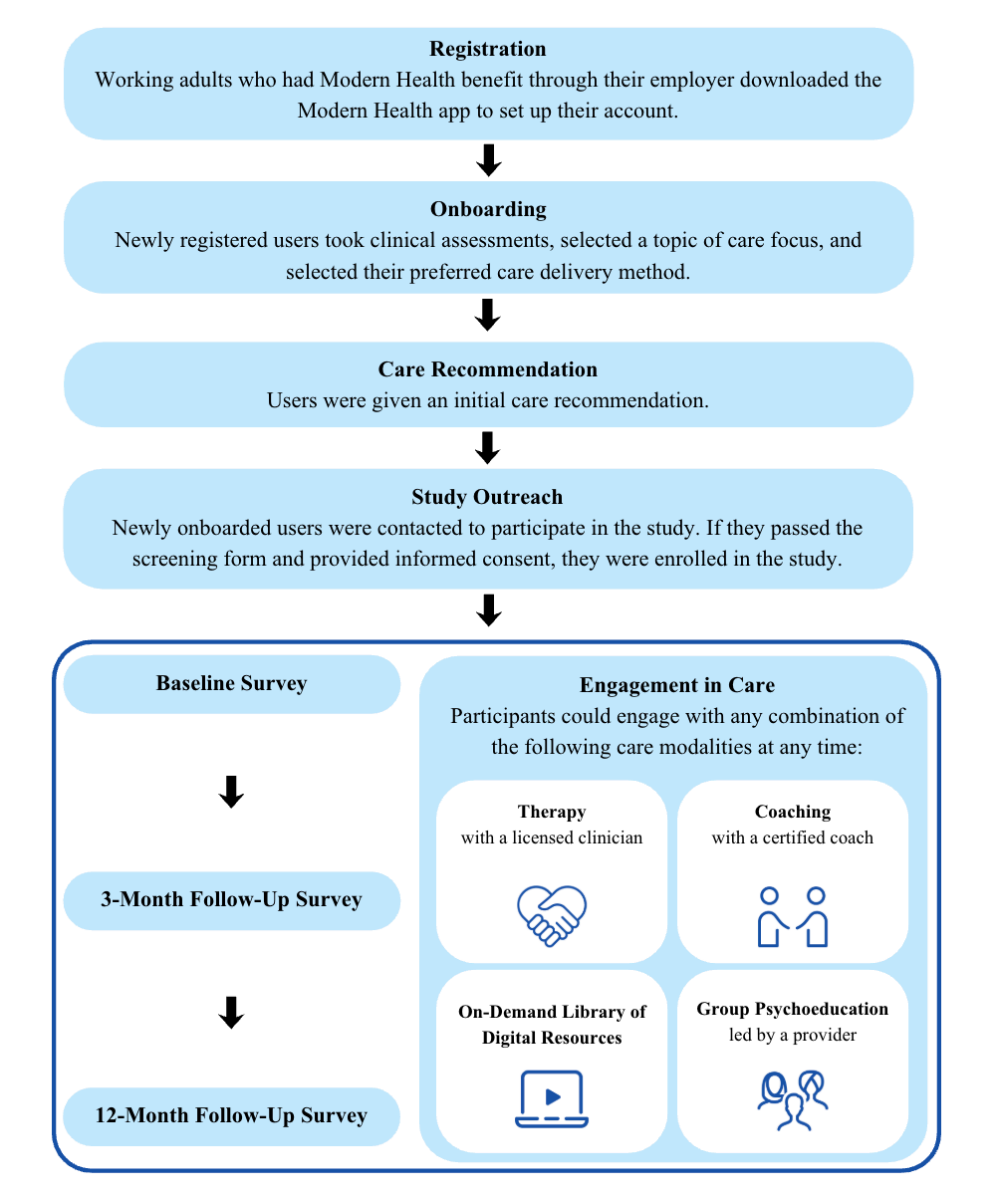
Flowchart of study intervention. Working adults who were newly registered and onboarded to the platform and met eligibility criteria were outreached for participation in the study. The baseline survey was sent to participants when they first initiated care, and after 3 and 12 months of using the platform services.

Eligible individuals registered for an account with the platform via a mobile app or website. During the onboarding process, users completed clinical assessments using validated measures such as the 9-item Patient Health Questionnaire (PHQ-9) and the 7-item Generalized Anxiety Disorder scale (GAD-7) to determine clinical need. Users also specified their preferred care delivery method, such as “on my own,” “one-on-one,” or “unsure,” and selected their desired area of focus from the following domains: emotions, physical well-being, professional life, relationships, and finance. From these selections, a proprietary algorithm provided them with an initial care recommendation (eg, to start with coaching for a member with mild clinical need who preferred one-on-one support). After onboarding to the platform, users could begin using the digital mental health services. Individuals were able to engage in services based on their care recommendation, or they could self-refer and use any combination of multimodal care, including one-on-one care, group psychoeducation, and self-guided resources as they desired. Upon completing the onboarding process, individuals who met the specified inclusion criteria were invited by email to fill out a screening questionnaire. This questionnaire gathered additional demographic information to ensure a balanced sample that was representative of the broader US population and the commercial population who had access to this employer-sponsored benefit. Those who successfully completed the screening were subsequently directed to a consent form. Participants who provided their informed consent to join the study received a baseline survey via email, which took approximately 30‐45 minutes to complete. Throughout the study period, participants engaged with the digital mental health services and received two follow-up surveys at 3 and 12 months via email. All questionnaires and surveys were conducted using Qualtrics’ online survey platform. The study procedure is depicted in Figure 1.

### Digital Mental Health Services

#### Overview

The digital mental health platform is an employer-sponsored mental health benefit, offering employees no-cost access to a range of mental health services such as one-on-one care with a therapist or coach, group psychoeducation sessions, and self-guided digital resources. For eligibility in this study, all participants accessed at least one therapy or coaching session, or engaged with at least one piece of digital content.

#### Therapy and Coaching

Participants had access to 50-minute one-on-one therapy sessions provided in person or via Zoom by licensed therapists holding advanced psychology or related degrees (eg, PhD, PsyD, LCSW, LMFT, or LPC). Additionally, participants had access to 30-minute coaching sessions provided via Zoom by International Coaching Federation–certified coaches [41]. All coaches had at least 100 hours of coaching experience plus at least 6 additional hours of training from the digital mental health company on topics of culturally centered care and identifying high-risk situations that may require crisis or higher-level support. All therapists and coaches were vetted carefully for offering evidence-based practices such as motivational interviewing, cognitive behavioral therapy, acceptance and commitment therapy, and dialectical behavior therapy. Ongoing provider quality monitoring was conducted by a team of legal, compliance, provider operations, and clinical experts to ensure rigorous clinical standards were upheld.

Participants were recommended to these one-on-one services after their initial intake assessment, or they could opt to self-refer. In both cases, a proprietary algorithm matched participants with a list of providers who were most relevant to their clinical needs, spoke their desired language, and had session availability within a few days. The matching algorithm also took into account provider specializations in the participant’s domain of focus. For example, if a participant wanted to work on their physical well-being and sleep quality, they were provided a list of therapists or coaches who specialized in sleep-related issues and could provide personalized strategies and support to help improve sleep habits. In this health domain, providers have the opportunity for individualized care, in which they could provide psychoeducation around how sleep and mental health are related, build routines around sleep hygiene and rest, implement techniques for cognitive restructuring to disrupt worrying and maladaptive thoughts at bedtime, and use motivational interviewing to support behavior changes necessary for sleep and mental health improvement. The amount and frequency of sessions that participants attended depended on their personal preferences, employer coverage, and individual needs.

#### Self-Guided Digital Resources and Group Psychoeducational Sessions

Participants had unrestricted access to an on-demand digital library featuring mental health programs and resources available to them. These included brief daily exercises, interactive programs, educational podcasts, mindfulness practices, and self-paced educational courses designed with the support of mental health experts including an in-house team of clinical psychologists, covering topics like emotions, relationships, and professional life. In addition to ample content aimed at improving mental health, the digital library also contained several resources specific to sleep. These included a self-paced, structured 6-session course called “Tools for Better Sleep, Rest, and Movement Throughout Your Day” and a 6-episode podcast covering elements of cognitive behavioral therapy for those with clinical insomnia (CBTi) including sleep hygiene, bedtime stimulus control, relaxation techniques, sleep restriction, and cognitive restructuring. There were also guided meditations, body scans, progressive muscle relaxations, breathing exercises, and calming sounds designed to help with sleep. Providers could recommend self-guided resources that were relevant to participants’ concerns or goals. Participant use of self-guided resources was recorded; however, specific content selection was not.

Finally, participants could join live group psychoeducation sessions led by therapists and coaches. Sessions covered topics such as dealing with challenges in the workplace, processing stressful world events, and building emotional resilience. Several regularly featured sessions were specific to improving sleep quality (eg, “Sleep: A Beginner’s Guide” and “Sleep: A Deep Dive,” both 1-hour-long sessions). Group sessions were provided in a variety of formats, such as webinar-style learning, skill-building workshops, and informal discussions to support different user preferences. Participation in the group sessions was anonymous.

### Measures

#### Demographics

Participants provided information on their age (by typing a number in years in an open-text box) and selected their gender identity, race/ethnic identity, and level of education from preset dropdown menus, which also had the option to include another category.

#### Sleep Quality

Perceived sleep quality was measured by a single item (“During the past month, how would you rate your sleep quality overall?”) from the Pittsburgh Sleep Quality Index [42]. Participants could choose 1 of 4 response options: (3) “very good,” (2) “fairly good,” (1) “fairly bad,” or (0) “very bad.” A binary variable was created, such that “very bad” or “fairly bad” responses were categorized as “poor sleep,” while “fairly good” or “very good” responses were categorized as “good sleep.” We defined improvement in sleep quality as a participant being categorized as having “poor sleep” at baseline and “good sleep” at a follow-up timepoint. Single-item self-rated health measures are increasingly being used in clinical practice and health research due to their feasibility and strong predictive validity [43,44]. In real-world settings, lengthy questionnaires are not always practical, especially when examined at multiple timepoints. As such, this single-item measure of sleep quality was chosen to capture a relatively accurate perception of one’s sleep. Single-item sleep quality measures have been used in previous studies [20,45,46], and research suggests that one’s perception of their sleep quality can be more predictive of well-being and daytime functioning than objective measures [37-39].

#### Depression

Depression severity was measured by the PHQ-9 [47]. Participants reported how often they were bothered by a number of depression symptoms over the past 2 weeks, on a 4-point scale (“0=not at all” to “3=nearly every day”), with higher total scores indicating greater symptomatology. A positive screen for depression symptoms was indicated by a score of ≥10 [47].

#### Anxiety

Anxiety severity was measured by the GAD-7 [48]. Participants reported how often they were bothered by a number of anxiety symptoms over the past 2 weeks, on a 4-point scale (“0=not at all” to “3=nearly every day”), with higher total scores indicating greater symptomatology. A positive screen for anxiety symptoms was indicated by a score of ≥8 [49].

#### Workplace Burnout

Burnout was measured by the 16-item Maslach Burnout Inventory (MBI-GS-16) [50], which examines 3 different subscales of burnout (exhaustion, cynicism, and professional efficacy). Participants reported how often they experienced a number of feelings about their job on a 7-point scale (“0=never” to “6=every day”). An average was taken from the total score of each subscale. Higher average scores on the exhaustion subscale indicate greater feelings of fatigue and emotional exhaustion from work. Higher average scores on the cynicism subscale indicate greater indifference toward one’s work. Higher average scores on the efficacy subscale indicate greater feelings of competence and achievement in one’s work; thus, lower scores on this subscale indicate higher burnout. Higher levels of burnout were categorized by a frequency of “once a week” or more for exhaustion and cynicism subscales (≥4), and a frequency of “a few times per month” or less for the efficacy subscale (≤3).

#### Platform Engagement

To assess engagement in the platform, we reported average utilization for coaching, therapy, and self-guided digital resources. We included participants in this study who had used at least one service during the study timeframe. We did not have access to data on group psychoeducation sessions engagement as participation was anonymous.

### Statistical Analyses

All analyses were conducted using R software (version 4.4.1; R Foundation for Statistical Computing). We conducted descriptive statistics for demographic variables, baseline sleep quality, mental health, and burnout. We also calculated the percent of participants categorized as having “poor sleep” or “good sleep” at baseline, as well as those screening positive for depression symptoms, anxiety symptoms, or high levels of burnout in the overall sample.

Our first aim was to evaluate whether baseline sleep quality was associated with mental health (depression and anxiety) and workplace burnout (exhaustion, cynicism, and professional efficacy). For this aim, chi-square tests examined whether there were differences in the proportions of positive screens for depression, anxiety, or high burnout between participants with poor sleep quality versus those with good sleep quality.

Our second aim was to evaluate longitudinal changes in sleep quality among employees engaging with the digital mental health platform across 3 and 12 months. First, we descriptively report the proportion of participants whose sleep quality improved (went from poor to good), maintained the same sleep quality, or worsened (went from good to poor) from baseline to follow-up timepoints.

Second, a generalized linear mixed-effects model with a logit link was conducted using the *lme4* package [51] to assess if time since registering for the mental health platform predicted the odds of reporting good sleep quality.


$$\log⟮\frac{P(Yit=1)}{\left(Yit=0\right)}⟯=\beta_{0}+\beta_{1}({Time}_{ij})+{Subject}_{i}$$

In this model, the outcome variable was sleep quality (coded as binary: 0=poor sleep and 1=good sleep). The model included a fixed-effect for time since registering for the mental health platform (baseline=0, 3-month follow-up=3, and 12-month follow-up=12) and a random intercept for participants to account for different baseline sleep quality levels. Overdispersion was assessed with Pearson residuals, with no evidence of overdispersion observed (dispersion ratio=0.46). Mixed-effects models were selected for our longitudinal analyses in aims 2 and 3 to examine changes across the 3 timepoints while accounting for repeated measures within participants and accounting for data missing to follow-up using maximum likelihood estimation, which assumes data are missing at random.

Our third aim was to evaluate whether changes in sleep quality over time (between baseline, 3-month follow-up, and 12-month follow-up) were associated with concurrent changes in mental health symptoms and burnout. First, we conducted a series of linear mixed-effects models with the overall sample to examine the relationship between sleep quality and outcomes over the course of accessing the digital mental health services. Five models were constructed, one for each of the 5 mental health and burnout outcomes, all of which were continuous.


$$Y_{it}=\beta_{0}+\beta_{1}{Time}_{it}+\beta_{2}{SleepQuality}_{it}+{Subject}_{i}+\epsilon_{it}$$

The models included fixed effects for time since registering for the mental health platform (baseline=0, 3-month follow-up=3, and 12-month follow-up=12) and sleep quality (coded as binary: 0=poor sleep and 1=good sleep). Random intercepts for time were included at the participant level to account for individual differences in baseline mental health or burnout levels. Diagnostics confirmed that model assumptions were adequately met. Quantile-quantile plots showed approximate normality of residuals and random effects, with only minor deviations at the tails. Residual plots showed no evidence of heteroscedasticity, with residuals evenly distributed across fitted values and no systematic patterns. Finally, models converged without warning. For the second part of aim 3, as an exploratory, descriptive analysis, we conducted paired samples *t* tests among participants who reported poor sleep quality at baseline and good sleep quality at the 3- or 12-month follow-ups, with the aim of describing symptom trajectories among individuals whose sleep improved. These subgroup analyses were intended to complement the full-sample longitudinal analyses. We calculated percent change scores in mean symptoms of depression, anxiety, and burnout between study timepoints using the formula: [(follow-up mean – baseline mean)/baseline mean] × 100.

## Results

### Study Participants and Baseline Descriptive Data

We outreached to 8786 users who were initially eligible for the study, of which 2032 completed the screening form, and 950 agreed to participate and provided their informed consent. Of the 950 who consented and completed the baseline survey, 734 completed at least one follow-up survey, 724 provided sleep data for at least one follow-up survey, and of those, 578 engaged with at least one service between their baseline and 12-month survey timepoints. Participants were, on average, 33.88 (SD 8.73) years old, 61.1% (353/578) identified as women, and 40.3% (233/578) identified as a person of color (see Table 1 for sample demographics and characteristics). The full analytic sample (N=578) was used in our baseline descriptive and cross-sectional analyses, as well as longitudinal analyses with mixed-effects models, to maximize use of available data across timepoints. Of this group, 527 participants engaged with the platform between baseline and 3-month follow-up and provided data at 3 months; this sample was used in the analyses reporting the descriptive proportions of change and paired *t* tests examining change from baseline to 3 months. Similarly, 343 participants engaged between baseline and 12-month follow-up and provided data at 12 months; this sample was used in analyses reporting the descriptive proportions of change and paired *t* tests examining change from baseline to 12 months.

**Table 1.** Baseline demographic characteristics and descriptive statistics in the overall sample and by baseline sleep quality.

Demographic characteristic	Overall sample (N=578)	Poor sleepers (n=243)	Good sleepers (n=335)
Age, mean (SD)	33.88 (8.73)	33.95 (8.36)	33.82 (9.0)
Gender identity, n (%)			
Woman	353 (61.1)	160 (65.8)	194 (57.9)
Man	197 (34.1)	74 (30.5)	124 (37.0)
Gender nonbinary	28 (4.8)	9 (3.7)	17 (5.1)
Race and ethnicity, n (%)			
Asian or Asian American	109 (18.9)	45 (18.5)	64 (19.1)
Black or African American	36 (6.2)	20 (8.2)	16 (4.8)
Hispanic, Latino, or Spanish Origin	47 (8.1)	20 (8.2)	27 (8.1)
Non-Hispanic White	345 (59.7)	138 (56.8)	207 (61.8)
More than 1 race/ethnicity	39 (6.7)	19 (7.8)	20 (6.0)
Education^a^, n (%)			
<Bachelors	72 (12.5)	40 (16.5)	32 (9.6)
Bachelors	348 (60.2)	141 (58.0)	207 (61.8)
>Bachelors	158 (27.3)	62 (25.5)	96 (28.7)
Platform engagement over 12 months, mean (SD)			
Therapy sessions	6.25 (5.42)	5.60 (4.37)	6.79 (6.15)
Coaching sessions	3.39 (3.10)	3.48 (3.32)	3.32 (2.96)
Digital resources	10.94 (18.40)	10.21 (13.78)	11.47 (21.14)

a Difference is statistically significant (*P*<.05).

At baseline, 42% (243/578) of participants indicated their sleep quality over the past month was either “very bad” (n=24) or “fairly bad” (n=219), categorizing them as baseline “poor sleepers.” The remaining 58% (335/578) of participants indicated their sleep quality as either “fairly good” (n=289) or “very good” (n=46), categorizing them as baseline “good sleepers.”

For mental health symptoms at baseline, average depression (mean 8.35, SD 5.63) and anxiety scores (mean 7.63, SD 5.37) were below the clinical cutoffs of ≥10 for the PHQ-9 and ≥8 for the GAD-7. Additionally, 36.9% (213/578) of participants had a positive baseline screen for depression symptoms, and 42.9% (248/578) of participants had a positive baseline screen for anxiety symptoms.

For burnout symptoms at baseline, average exhaustion (mean 2.08, SD 1.67) and cynicism (mean 2.03, SD 1.59) were below the high burnout cutoffs of ≥4 for high exhaustion and cynicism on the MBI-GS-16. Average professional efficacy (mean 4.58, SD 1.05) was above the high burnout cutoff of ≤3 for low professional efficacy. Additionally, 26.9% (155/576) of participants were above the cutoff indicating high exhaustion, 16.1% (93/577) of participants were above the cutoff indicating high cynicism, and 8.7% (50/575) of participants were below the cutoff indicating low professional efficacy.

Regarding platform engagement during the 12-month study period, 42.9% (147/343) used therapy, attending an average of 6.25 virtual therapy sessions, 63.6% (218/343) used coaching, attending an average of 3.39 virtual sessions, and 77.3% (265/343) accessed self-guided digital content, engaging with an average of 10.94 resources. The majority of participants (221/343, 64.4%) used at least 2 modalities of care, and 19.2% (66/343) used all 3 modalities of care at least once.

### Baseline Associations Between Sleep Quality, Mental Health, and Burnout

Average depression and anxiety scores were below clinical cutoffs for those with good sleep quality (depression: mean 6.18, SD 4.73; anxiety: mean 6.03, SD 4.62) and above clinical cutoffs for those with poor sleep quality (depression: mean 11.30, SD 5.41; anxiety: mean 9.82, SD 5.57). Chi-squares indicated a higher proportion of participants with poor sleep at baseline screened positive for depression [*χ*^2^_1_(N=578)=102.47, *P*<.001, *ϕ*=0.42] and anxiety [*χ*^2^_1_(N=578)=61.97, *P*<.001, *ϕ*=0.33] than those with good sleep at baseline (see Table 2). For example, 60.9% (148/243) of poor sleepers screened positive for depression at baseline versus 19.4% (65/335) of good sleepers.

**Table 2.** Positive screens for mental health and high burnout in the overall sample and by baseline sleep quality^a^.

	Overall sample (N=578)	Poor sleepers (n=243)	Good sleepers (n=335)
Mental health symptoms			
Depression screen^b^, n (%)			
Negative screen	365 (63.2)	95 (39.1)	270 (80.6)
Positive screen	213 (36.9)	148 (60.9)	65 (19.4)
Anxiety screen^b^, n (%)			
Negative screen	330 (57.1)	92 (37.9)	238 (71.0)
Positive screen	248 (42.9)	151 (62.1)	97 (29.0)
Burnout symptoms			
High exhaustion^b^, n (%)			
Negative screen	421 (73.1)	151 (62.4)	270 (80.8)
Positive screen	155 (26.9)	91 (37.6)	64 (19.2)
High cynicism^b^, n (%)			
Negative screen	484 (83.9)	191 (78.6)	293 (87.7)
Positive screen	93 (16.1)	52 (21.4)	41 (12.3)
Low professional efficacy^b^, n (%)			
Negative screen	525 (91.3)	212 (87.2)	313 (94.3)
Positive screen	50 (8.7)	31 (12.8)	19 (5.7)

a A positive screen for depression or anxiety was defined as baseline depression or anxiety scores above the clinical cutoff (≥10 for depression, ≥8 for anxiety). High burnout was defined as exhaustion and cynicism scores ≥4, and professional efficacy scores ≤3.

b The difference is statistically significant (*P*<.05).

Average burnout exhaustion and cynicism were below the high burnout thresholds for both individuals with both good sleep quality (exhaustion: mean 2.49, SD 1.59; cynicism: mean 1.84, SD 1.50) and poor sleep quality (exhaustion: mean 3.23, SD 1.69; cynicism: mean 2.28, SD 1.68). Additionally, average professional efficacy was above the low professional efficacy threshold for individuals with both good sleep quality (mean 4.72, SD 0.99) and poor sleep quality (mean 4.4, SD 1.1). However, chi-squares indicated a higher proportion of participants with poor sleep at baseline had high burnout for exhaustion [*χ*^2^_1_(n=576)=23.34, *P*<.001, *ϕ*=0.2], cynicism [*χ*^2^_1_(n=577)=8, *P*=.005, *ϕ*=0.12], and low professional efficacy [*χ*^2^_1_(n=575)=7.88, *P*=.005, *ϕ*=0.12] than those with good sleep at baseline (see Table 2). For example, 37.6% (91/242) of poor sleepers were classified as having high exhaustion at baseline versus 19.2% (64/334) of good sleepers.

### Longitudinal Changes in Sleep Quality

Descriptively, the proportion of participants categorized as poor sleepers was the following at each timepoint: 42% (243/578) at baseline, 38.1% (201/527) at 3 months, and 35.9% (123/343) at 12 months. From baseline to 3 months, 81.8% (248/303) of participants who reported good sleep at baseline maintained good sleep quality at 3 months, while 18.2% (55/303) worsened and reported poor sleep quality at follow-up. Additionally, 65.2% (146/224) of participants who reported poor sleep at baseline maintained poor sleep quality at 3 months, while 34.8% (78/224) improved and reported good sleep quality at follow-up. From baseline to 12 months, 78.2% (158/202) of participants who reported good sleep at baseline maintained good sleep quality at 12 months, while 21.8% (44/202) worsened and reported poor sleep quality at follow-up. Additionally, 56% (79/141) of participants who reported poor sleep at baseline maintained poor sleep quality at 12 months, while 44% (62/141) improved and reported good sleep quality at follow-up.

**Figure 2.**
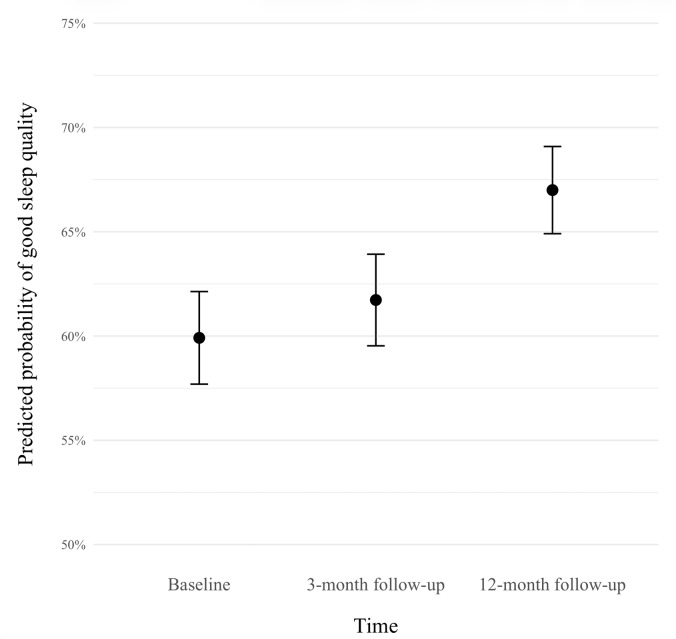
Predicted probability of good sleep quality by timepoint. The predicted probability of good sleep quality is 59.9% at baseline, 61.7% at 3-month follow-up, and 67% at 12-month follow-up. Error bars represent 95% CIs.

A generalized linear mixed-effects model was constructed to predict the odds of good sleep quality over time (see Figure 2). The model included a fixed effect of time and random intercept for participants. The model indicated that time since registering for the mental health platform had a small but statistically significant association with sleep quality (β=0.04, SE=0.02; *z*=2.29, two-tailed; *P*=.02). The β of 0.037 for time translates to an odds ratio of 1.037 (95% CI 1.006-1.071), indicating that for each additional month of platform access, the predicted odds of having good sleep quality increased by 3.7%. The model predicted that compared to baseline, the odds of having good sleep quality were 11.6% higher at 3 months and 55.1% higher at 12 months. Based on this model, Figure 2 depicts the predicted probability of having good sleep quality at each timepoint.

### Relationships Between Sleep Quality, Mental Health, and Burnout Over Time

A series of linear mixed-effects models (see Table 3) were constructed to examine the impact of time since initiation of platform services and sleep quality on 5 mental health and burnout outcomes across the 3 study timepoints. In the model predicting depression, time since registering for the platform was significantly associated with depression symptom reduction (β=−0.16, *P*<.001). Sleep quality significantly predicted depression symptoms when controlling for time (β=−3.7, *P*<.001), such that a 1 unit increase in sleep quality (moving from poor sleep to good sleep) was associated with a 3.7 point reduction in depression scores. Given the intercept was 9.81 (close to the cutoff of ≥10 for a positive depression screen), a 3.7 point reduction associated with improved sleep quality indicates that average depression scores reduced to 6.11, which are approaching the mild range (<5). Similarly, for anxiety, time since registering for the platform was significantly associated with anxiety symptom reduction (β=−0.12, *P*<.001). Sleep quality significantly predicted anxiety symptoms when controlling for time (β=−2.9, *P*<.001), such that a 1 unit increase in sleep quality was associated with a 2.9 point reduction in anxiety scores. The intercept in this model was 8.89 (above the cutoff of ≥8 for a positive anxiety screen); thus, a 2.9 point reduction associated with improved sleep quality indicates average anxiety scores reduced to 5.99, which are approaching the mild range (<5).

**Table 3.** Fixed effects estimates of time and sleep quality on mental health and burnout outcomes^a^.

Outcome variable and predictor	β	SE	*t* test^b^ (*df*)	*P* value
Mental health				
Depression				
(Intercept)	9.81	0.22	43.88 (1159.75)	<.001
Time	–0.16	0.02	–7.72 (980.77)	<.001
Sleep quality	–3.70	0.25	–15.07 (1467.82)	<.001
Anxiety				
(Intercept)	8.89	0.23	37.87 (1149.47)	<.001
Time	–0.12	0.02	–5.71 (967.75)	<.001
Sleep quality	–2.90	0.25	–11.54 (1467.06)	<.001
Burnout				
Exhaustion				
(Intercept)	3.07	0.08	39.11 (1063.65)	<.001
Time	0.04	0.01	6.74 (924.95)	<.001
Sleep quality	–0.50	0.08	–6.60 (1326.02)	<.001
Cynicism				
(Intercept)	2.23	0.08	28.40 (1109.20)	<.001
Time	0.07	0.01	11.09 (941.18)	<.001
Sleep quality	–0.30	0.08	–3.70 (1400.73)	<.001
Professional efficacy				
(Intercept)	4.43	0.05	85.99 (1122.00)	<.001
Time	–0.01	0.00	–3.19 (948.96)	.002
Sleep quality	0.22	0.05	4.11 (1417.82)	<.001

a Time is coded as 0 for baseline, 3 for 3-month follow-up, and 12 for 12-month follow-up. Sleep quality is coded as 0 for “poor sleep” and 1 for “good sleep.”

b All tests are 2-tailed.

In the model predicting burnout exhaustion, time since registering for the platform was significantly associated with an exhaustion score increase (β=0.04, *P*<.001). Sleep quality significantly predicted exhaustion when controlling for time (β=−0.5, *P*<.001), such that a 1 unit increase in sleep quality was associated with a 0.5 point reduction in exhaustion scores. For cynicism, time since registering for the platform was significantly associated with a cynicism score increase (β=0.07, *P*<.001). Sleep quality significantly predicted cynicism when controlling for time (β=−0.3, *P*<.001), such that a 1 unit increase in sleep quality was associated with a 0.3 point reduction in cynicism scores. For professional efficacy, time since registration for the platform was significantly associated with an efficacy score decrease (β=−0.01, *P*=.002). Sleep quality significantly predicted professional efficacy when controlling for time (β=0.22, *P*<.001), such that a 1 unit increase in sleep quality was associated with a 0.22 increase in professional efficacy scores.

Next, we conducted paired sample *t* tests within the subset of participants who improved their sleep from baseline to 3- or 12-month follow-ups (Table 4) to better understand how mental health symptoms and burnout changed between the study timepoints (from baseline to 3-month, or from baseline to 12-month). Participants with poor sleep at baseline who reported good sleep at 3-month follow-up (n=78) showed significant reductions in depression (−42.8%) and anxiety (−30.5%; all *P*<.001) between baseline and 3-month follow-up. Participants with poor sleep at baseline who reported good sleep at 12-month follow-up (n=62) showed significant reductions in depression (−48.3%) and anxiety (−38.3%; all *P*<.001) at 12-month follow-up. None of the 3 burnout domains changed over 3 or 12 months in the samples of participants who improved their sleep quality at 3- or 12-month follow-up, with the exception of a significant increase in cynicism at 12-month follow-up (23.9%, *P*<.01), though it remained below the cutoff for high burnout.

**Table 4.** Paired sample *t* tests of outcomes among participants who improved their sleep from baseline to follow-up timepoints.

Variable	n	Range	Baseline, mean (SD)	Follow-up, mean (SD)	*t* test^a^ (*df*)	*P* value	Cohen *d*
Baseline to 3-month follow-up							
Depression	78	0‐27	10.17 (5.29)	5.82 (4.10)	–7.05 (77)	<.001	0.80
Anxiety	78	0‐21	8.94 (5.94)	6.21 (4.70)	–4.86 (77)	<.001	0.55
Exhaustion	78	0‐6	2.93 (1.75)	2.85 (1.79)	–0.51 (77)	.61	0.06
Cynicism	78	0‐6	2.17 (1.61)	2.33 (1.77)	1.14 (77)	.26	0.13
Efficacy	78	0‐6	4.53 (1.11)	4.54 (1.06)	0.05 (77)	.96	0.01
Baseline to 12-month follow-up							
Depression	62	0‐27	11.40 (5.96)	5.90 (4.21)	–7.35 (61)	<.001	0.93
Anxiety	62	0‐21	9.60 (5.92)	5.92 (4.91)	–5.55 (61)	<.001	0.70
Exhaustion	58	0‐6	3.26 (1.70)	3.06 (1.46)	–0.54 (57)	.59	0.07
Cynicism	59	0‐6	2.38 (1.74)	2.95 (1.64)	2.55 (58)	.01	0.33
Efficacy	59	0‐6	4.39 (1.03)	4.40 (1.05)	0.28 (58)	.78	0.04

a All tests are 2-tailed.

## Discussion

### Main Findings

To date, sleep research has primarily focused on the treatment of insomnia instead of taking a broader public health perspective to sleep treatment. Given the impact of sleep perception on mental health and well-being outcomes, a broader focus on sleep among the general population may offer critical insights into public health interventions to address sleep. We evaluated the real-world effectiveness of an employer-sponsored digital mental health platform for improving sleep quality and also evaluated whether improved sleep quality was associated with changes in mental health and burnout. As predicted, we found that engagement with the mental health platform was associated with improved sleep quality. Additionally, when sleep quality was higher, individuals tended to show reduced mental health and burnout symptoms. This study is important as it assessed the impact of a multimodal mental health platform within a broad population.

The large percentage of participants who registered for the mental health platform who reported poor sleep quality at baseline (243/578, 42%) supports a plethora of research showing that many people struggle to achieve restful and restorative sleep at night. This finding mirrors an American Psychological Association statistic from 2013, where 42% of adults reported fair or poor quality sleep [20]. For such individuals, especially those who experience sleep issues but do not reach the threshold for a sleep disorder diagnosis, there is a clear need for services that can help to improve sleep problems. Our findings suggest that multimodal digital mental health platforms, though not explicitly a sleep intervention, can offer support beyond just mental health, playing a valuable role in other health behaviors and domains of well-being known to influence mental health.

When examining correlates of poor sleep before starting care, we found that individuals with poor sleep quality at baseline were more likely to report higher depression and anxiety symptoms, greater exhaustion and cynicism at work, and lower professional efficacy for their job. These associations align with past research showing that maintaining quality sleep is crucial for sustaining the cognitive resources needed to tackle daily challenges [15,17] and managing negative emotional experiences [5,18]. As such, sleep disturbances may make it difficult to manage and recover from daily challenges, leading to exhaustion, feelings of burnout, and greater mental health symptoms. Given the bidirectional relationship between these variables, the opposite may also be true. Experiencing greater mental health and workplace concerns could lead to greater sleep disturbances. Individuals may lie awake at night with intrusive thoughts or worry about work-related challenges [20-22]. Additionally, the body’s physiological response to stressors, such as greater cortisol in the body, can disrupt the wake-sleep cycle [52]. While this study cannot determine the directionality of these baseline relationships, our findings strongly underscore the complex interplay between these variables. Given that the population studied here was seeking mental health support from a digital platform, these baseline findings highlight the range of health and well-being needs of these individuals beyond care for depression or anxiety.

Although the services in the digital mental health platform primarily focused on emotional health, the platform also leveraged multiple modalities of care (therapy, coaching, and self-guided resources) to support people with their physical well-being and sleep habits. Thus, for aim 2, we explored whether sleep quality improved over the course of using digital mental health services. We found that 34.8% (78/224) of participants who initially reported poor sleep at baseline improved to good sleep by the 3-month follow-up, and 44% (62/141) improved to good sleep by the 12-month follow-up. Additionally, our model examining change in sleep quality across all 3 timepoints, while accounting for repeated measurement within participants and missing data, predicted that the odds of reporting good sleep quality consistently increased over time. Specifically, the odds of experiencing good sleep quality rose by 3.7% for each month of platform access, indicating a 55.1% increased odds of good sleep after 12 months. While not everyone experienced sleep improvements, and some individuals experienced worsening sleep symptoms over time, sleep issues can be difficult to change for some individuals. Future research may explore which individuals are less likely to experience sleep improvement and the factors underlying this resistance to change. Nonetheless, the overall findings are promising for this type of innovative intervention, and in line with previous research showing that mental health treatment can also lead to welcomed improvement in sleep quality [29,30,40].

The improvement in sleep quality observed in our study underscores the potential for digital mental health interventions to support sleep-related concerns. These platforms provide a unique opportunity to reach individuals with subclinical sleep issues or those who may not present or have access to sleep treatments like CBTi. Given the high rate of sleep concerns in the general public, sleep problems have become somewhat normalized. As a result, many individuals may overlook these concerns, fail to seek support for their sleep outside of the traditional medical system, or face barriers when they do [53]. Additionally, gold standard interventions like CBTi can be time intensive, and research on these interventions has focused primarily on clinical populations. This leaves a gap in our understanding of the impact of less intensive, but still evidence-grounded, interventions that could have broad public health implications. Digital mental health platforms can serve as an accessible entry point for sleep support, offering an alternative to standard treatments for sleep that may be less accessible, limited in availability, or not well-known. As services for these mental health platforms are carefully developed, they may consider taking a holistic care approach to support the diverse and interconnected health and well-being needs of their users.

Given the strong link between sleep quality, mental health, and burnout, for aim 3, we explored whether changes in sleep quality over time were associated with changes in depression, anxiety, and all 3 domains of burnout. Between our 2 analyses, we found that improved sleep quality was associated with improved mental health symptoms and, to some extent, workplace burnout. Examining mental health across time, our regression models predicted a significant reduction in depression and anxiety symptoms. Additionally, when one’s sleep quality was good, depression and anxiety were significantly lower by 3.7 points and 2.9 points, respectively. These models showed that at baseline, depression and anxiety were close to or above the cutoffs for a positive screen and moderate symptoms, and that when sleep quality improved, average scores dropped to around 6, which is closer to mild levels of symptoms.

In our second analysis for this aim, we found that among participants who reported poor sleep at baseline and improved to good sleep by the 3-month follow-up, their average depression scores statistically significantly improved by 42.8%. This change of 4.35 points was also close to the threshold to be considered clinically significant change, which is a 5-point reduction in depression [54]. At 12 months, people who improved their sleep quality reported a 48.3% improvement in depression and a clinically significant point reduction of 5.5 points, on average. Regarding anxiety, those who improved their sleep quality by 3 months reported a 30.5% improvement at the 3-month follow-up, and those who improved their sleep quality by 12 months reported a 38.3% improvement in symptoms at the 12-month follow-up. It is possible that mental health improvements directly contributed to individuals experiencing better sleep quality. Alternatively, as the relationship between sleep and mental health is bidirectional [55], improvements in sleep quality may have had positive downstream effects on mental health. The digital mental health platform provided comprehensive services aimed at enhancing overall well-being, including physical health, with some resources specific to sleep hygiene and psychoeducation. Improvement in either sleep quality or mental health likely contributed to positive change in the other.

Regarding the association between changes in sleep quality and changes in the 3 domains of burnout, we first found that over time, exhaustion and cynicism significantly increased while professional efficacy decreased. However, burnout scores were already low at baseline, and this amount of change was not enough to move scores over the threshold to be considered high burnout at any timepoint. Sleep quality seemed to act as a kind of buffer against these increases. When one’s sleep quality was good, exhaustion and cynicism were statistically significantly lower by 0.5 points and 0.3 points, respectively, and professional efficacy was higher by 0.22 points. These point reductions in exhaustion and cynicism and increase in efficacy associated with good sleep quality across study timepoints put participants’ burnout scores even further into the category of lower burnout.

The buffering impact of good sleep quality was also partially supported by our exploratory paired sample *t* test analyses. While the regression analyses showed that burnout slightly increased over time, our *t* tests indicated that the subsample of participants who improved their sleep quality from baseline to either follow-up timepoint maintained their low levels of burnout. Directionally, exhaustion scores were lower at follow-up (though not statistically significant), cynicism scores were higher at follow-up (and significantly so at 12 months, but remained below the high burnout cutoff), and professional efficacy did not change (directionally or statistically). Because burnout was low at baseline, and remained below cutoffs throughout the study, there was limited room for further reduction. The slight increase in cynicism between baseline and 12 months may reflect a floor effect. However, the overall observed maintenance of low burnout over time, especially for those with good and improving sleep quality, is a positive outcome.

The study findings related to burnout may reflect additional contributing factors. Burnout may be attributed to aspects of organizational culture, job demands, and workplace policies [56,57] that remain unchanged despite individual improvements in sleep and mental health. This could account for the fact that burnout slightly declined over time, yet sleep quality and mental health tended to improve. It is also possible that burnout has a larger influence on sleep quality than the reverse. Individuals experiencing burnout may be facing work-related stressors such as working long hours and dealing with emotional exhaustion, which can disrupt their ability to maintain a consistent bedtime routine or mentally wind down before bed [25]. As burnout decreases, sleep quality may improve from the reduced stress and demands. Of note, only a small portion of participants had elevated burnout at baseline, and we did not stratify the sample by baseline burnout severity. It is possible that individuals with higher initial burnout experienced improvements in sleep quality as their burnout decreased. For the majority of participants who started out with low levels of burnout, their treatment goal may have been to maintain their lower levels, a pattern that aligns with the study findings.

Improvements in sleep alone may not address the organizational factors that likely have a more significant impact on burnout. The complex nature of burnout may require more targeted interventions, starting in the workplace itself with strategies such as systemic improvements, to see significant changes. However, improving sleep quality may still have a positive impact. Overall, burnout tended to be lower at time points when individuals reported good sleep quality. When sleep improved from poor to good, burnout levels remained low. Improving sleep through mental health interventions may help to buffer against the exacerbation of workplace stress and burnout. Therefore, it is worth investing in solutions that target sleep and mental health, as they can also affect how someone shows up at work, even when burnout may be increasing due to other organizational factors; improved sleep may attenuate this increase.

### Limitations

There are several notable limitations of the study. First, this was an observational study of real-world data collected between 2021 and 2023. As the study examined an existing digital mental health benefit, the study did not include a control group for comparison. Therefore, we are unable to make causal claims that the digital mental health platform caused the observed changes in sleep quality or mental health. Additionally, the aim 3 exploratory subgroup analysis focused on individuals whose sleep improved and was not designed to infer causality or compare outcomes with those whose sleep did not improve. Observed changes of these variables over time may have been related to a number of alternative factors, such as world events like the COVID-19 pandemic, life events, other temporal factors, or even regression to the mean due to the multiple time points accessed. However, several studies have demonstrated improvements in mental health following use of this digital mental health platform [58-60], and there is mounting evidence that individuals often experience enhanced sleep quality following improvements in their mental health [29,30,40]. Nonetheless, future studies could incorporate a comparison group in their design to further validate the effectiveness of digital mental health services in improving sleep.

Participants were eligible for the study if they attended at least one therapy or coaching session, or engaged with at least one self-guided digital resource. Many of our participants used a combination of one-on-one care and self-guided resources. Therefore, we were unable to stratify sleep quality improvements by care modality. Additionally, detailed information regarding the specific timing and duration of platform engagement was not available to the researchers. Future studies may explore how factors such as service type, content, level of engagement, and timing of engagement may influence the magnitude of sleep quality improvement. Future research may also consider using clustering approaches to identify distinct patterns of multimodal engagement across care types and examine how these patterns relate to mental health and sleep outcomes. This could help determine the most effective strategies for improving sleep quality with digital mental health services.

All participants were employees of organizations sponsoring the digital mental health platform. Therefore, the results of the study may not generalize to nonworking adults or employees that were not represented on the platform. For instance, factors such as job-related workload or financial stress may have impacted sleep quality, and these particular stressors may vary in other populations.

Finally, all data collected in this study were self-reported by participants, and individuals may not have accurately evaluated themselves or remembered their experiences. Furthermore, we used a single item to measure sleep quality perception. We did not assess objective measures of sleep quality, such as actual sleep duration, latency, and efficiency. However, research has shown that one’s perception of their sleep quality can strongly influence emotional well-being and cognitive performance, sometimes even more so than objective sleep metrics [37-39].

### Conclusions

This study examined how the use of an employer-sponsored digital mental health platform impacted perceived sleep quality and correlates of poor sleep over time. At baseline, poor sleep was related to higher symptoms of depression, anxiety, and burnout, highlighting the multifaceted nature of challenges experienced by a sample of participants motivated to engage in mental health care and support. Results suggest that digital mental health solutions offering a comprehensive, holistic approach to well-being can support sleep improvement. Sleep quality improved while participants engaged with services, and this improvement in sleep was associated with improvements in mental health symptoms and maintenance of burnout below cutoff levels. Given the strong connection between sleep and emotional well-being, the study demonstrates that sleep and mental health can be addressed simultaneously with digital mental health services, with improvements supporting other positive outcomes such as management of workplace burnout.

## Abbreviations

CBTi: cognitive behavioral therapy for those with clinical insomnia
d-CBTi: digital cognitive behavioral therapy for those with clinical insomnia
GAD-7: 7-item Generalized Anxiety Disorder scale
MBI-GS-16: 16-item Maslach Burnout Inventory
PHQ-9: 9-item Patient Health Questionnaire
